# E-DNA scaffold sensors and the reagentless, single-step, measurement of HIV-diagnostic antibodies in human serum

**DOI:** 10.1038/s41378-019-0119-5

**Published:** 2020-03-23

**Authors:** Claudio Parolo, Ava S. Greenwood, Nathan E. Ogden, Di Kang, Chase Hawes, Gabriel Ortega, Netzahualcóyotl Arroyo-Currás, Kevin W. Plaxco

**Affiliations:** 1 Department of Chemistry and Biochemistry, University of California, Santa Barbara, Santa Barbara, CA USA; 2 Department of Materials, University of California, Santa Barbara, Santa Barbara, CA USA; 3 Department of Pharmacology and Molecular Sciences, Johns Hopkins School of Medicine, Baltimore, MD 21205 USA; 4 Interdepartmental Program in Biomolecular Science and Engineering, University of California, Santa Barbara, Santa Barbara, CA 93106 USA

**Keywords:** Chemistry, Biosensors

## Abstract

The multiplexed, point-of-care measurement of specific antibodies could improve the speed with which diseases are diagnosed and their treatment initiated. To this end, we are developing E-DNA scaffold sensors, which consist of a rigid, nucleic acid “scaffold” attached on one end to an electrode and presenting both a redox reporter and an epitope on the other. In the absence of antibody, the reporter efficiently transfers electrons when interrogated electrochemically. Binding-induced steric hindrance limits movement, reducing electron transfer in a manner that is both easily measured and quantitatively related to target concentration. Previously we have used monoclonal antibodies to explore the analytical performance of E-DNA sensors, showing that they support the rapid, single-step, quantitative detection of multiple antibodies in small volume samples. Here, in contrast, we employ authentic human samples to better explore the platform’s clinical potential. Specifically, we developed E-DNA sensors targeting three HIV-specific antibodies and then compared the analytical and clinical performance of these against those of gold standard serological techniques. Doing so we find that, although the multistep amplification of an ELISA leads to a lower detection limits, the clinical sensitivity of ELISAs, E-DNA sensors and lateral-flow dipsticks are indistinguishable across our test set. It thus appears that, by merging the quantitation and multiplexing of ELISAs with the convenience and speed of dipsticks, E-DNA scaffold sensors could significantly improve on current serological practice.

## Sensors: detecting HIV antibodies

A single-step, electrochemical-based device has the ability to detect and measure the amount of HIV antibodies in clinical samples. There is an ever-growing need for tools that can diagnose diseases accurately, quickly and at point-of-care. The group of Kevin Plaxco at University of California Santa Barbara has previously demonstrated an E-DNA scaffold sensor for quantifying antibodies, but it had not been tested on real clinical samples. Now, they demonstrate that it can detect several antibodies for the diagnosis of HIV in clinical samples, and benchmark it against traditional gold standard techniques. The E-DNA sensor achieves 90% sensitivity and 100% specificity for the tested antibodies, values identical to those obtained using standard ELISA and lateral flow immunoassays when challenged with the same sample set.

## Graphical abstract

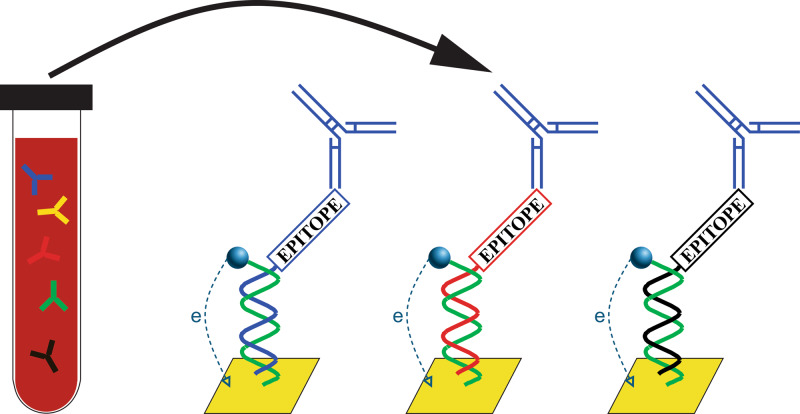

## Introduction

Although antibodies are perhaps the broadest and most important class of diagnostic biomarkers, our ability to measure (as opposed to simply detect the presence of) them at the point of care remains limited^1,2^. The current gold standard for antibody quantification, the enzyme-linked immunosorbent assay (ELISA), is a slow, cumbersome, laboratory-based technique that provides excellent analytical performance and is easily parallelized, but requires hours to deliver a result and is reliant on specialized personnel and equipment^3^. In contrast, the ease of use and low cost of lateral flow immunoassays renders them the undisputed leaders for antibody detection at the point of care^4,5^, but their qualitative nature and limited multiplexing reduces their utility^6,7^. A technology that combines the quantitative output and easy multiplexing of laboratory-based assays with the speed and single-step convenience of point-of-care testing could thus significantly augment current serological technologies, enabling improved diagnosis within the timeframe of a single visit to the clinic^8–11^.

**Fig. 1:**
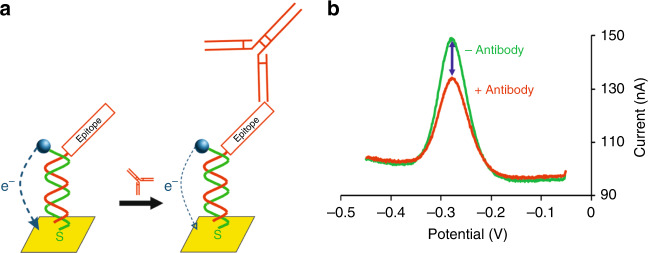
The E-DNA scaffold sensor supports the single-step measurement of the concentration of specific antibodies. **a** In the absence of the targeted antibody, the DNA scaffold efficiently transfers electrons to the gold electrode. Upon antibody binding, in contrast, steric hindrance reduces electron transfer. **b** The resultant change in electron transfer rate is easily detected using square wave voltammetry. As shown, for example, the addition of 10 nM of the target antibody (here an anti-FLAG antibody) produces a 30% decrease in the signaling current.

In recent years we have described a reagentless, single-step electrochemical approach to quantify antibodies, termed E-DNA scaffold sensor, that is not only quantitative and easily multiplexed but is also rapid (<10 min) and convenient (few operator steps) enough for deployment at the point of care^12^. The platform consists of a gold electrode modified with a DNA strand that presents a redox reporter on its 3′-end, which generates an electrochemical signal, and a thiol group on its 5′-end, which anchors the DNA to the gold surface (Fig. 1). The hybridization of a complementary oligonucleotide that presents on its distal end an antibody-binding epitope (recognition element) creates rigid, double-helical “scaffold” that is attached to the electrode surface by a flexible linker. The flexibility of the linker allows the redox reporter to approach the electrode, enhancing electron transfer. Upon binding, however, the steric bulk of the antibody limits this motion^13^, reducing electron transfer and producing a signal that is quantitatively related to the concentration of the target.

Previously we have characterized the analytical performance of the E-DNA scaffold sensor platform using a number of purified monoclonal antibodies^12,14^. We have likewise explored the maximum size antigen that it can successfully present^13^. But we had not previously characterized its potential clinical performance using clinical samples. To do so, here we have expanded the platform to the detection of several antibodies diagnostic for HIV, and then used a test set of HIV-positive and -negative human sera to characterize the new sensors’ clinical sensitivity and specificity relative to those of commercial ELISA and lateral flow immunoassays.

## Results

**Fig. 2:**
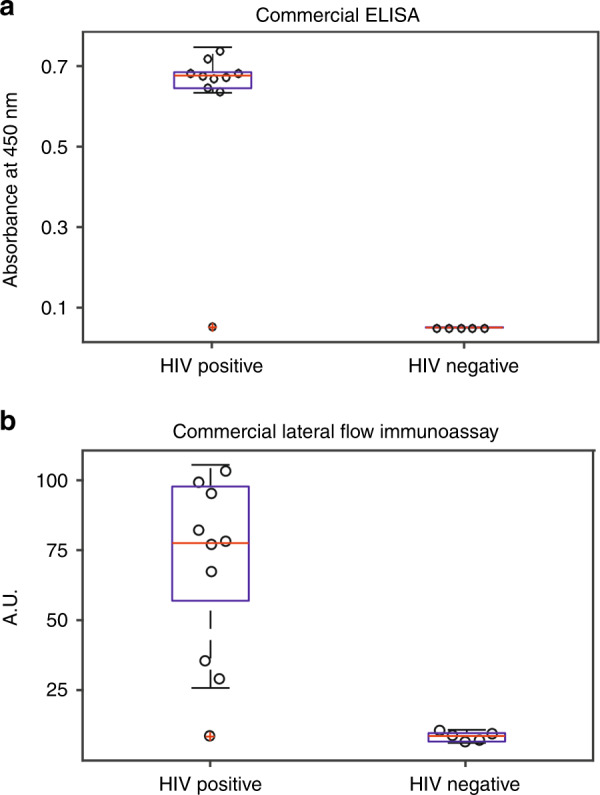
To establish “ground-truth” we analyzed 15 authentic patient samples using two established commercial tests. We tested ten (reportedly) HIV-positive and five (reportedly) HIV-negative commercially sourced human serum samples using **a** a commercial ELISA and **b** a commercial lateral flow immunoassay. Both achieved 90% clinical sensitivity (both flagged the same reportedly HIV-positive sample as HIV-negative—red dot) and 100% clinical specificity (both correctly determined all five HIV-negative samples to be negative). We quantified the ELISA results measuring the absorption at a wavelength of 450 nm, while lateral flow immunoassays using the “Analyze/Plot Profile” function in the ImageJ Program^16^.

To compare the performances of E-DNA scaffold sensors with that of current gold standard serological tests, we employed a set of 15 anonymized, commercially sourced human serum samples, 10 of which were putatively HIV-positive and 5 of which were putatively HIV-negative. Using these to test a commercially available ELISA (HIV-1/2 Ab ELISA Kit) and a commercially available lateral flow immunoassay (UniGold™ Recombigen® HIV-1/2), we found that both achieved 90% sensitivity (both tests flagged the same putatively HIV-positive patient—UID: 200760—as negative, leaving us to suspect that it is, counter to the provider’s claims, negative) and 100% specificity (Fig. 2). Looking at performance from the user perspective, the ELISA is far slower (>2 h), more cumbersome (it requires more than 20 steps for the detection, including buffer preparation, pipetting, multiple washes and incubations), and resource intensive (it requires a bulky, expensive plate reader for the quantification of the results). Conversely, it can support approximately 90 assays in parallel. Lateral flow immunoassays, in contrast, require merely two steps (the addition of sample followed by the addition of a washing buffer) and provide a result within 10 min. This said, lateral flow assays are qualitative, limiting the information they provide regarding the stage of the infection and reducing their specificity (the visual interpretation of faint bands can easily lead to misinterpretation of the results)^15^. They are also somewhat “finicky,” as the test must be read in a narrow time window (between 10 and 12 min after application); this can be an issue in busy clinical environments where a small delay may lead to a false reading. Given this, the bar for a new technology, such as the E-DNA platform, is to achieve 90% sensitivity and 100% specificity in this same sample set, in quantitative detail, in 2 or fewer steps, in less than 12 min, and without requiring a fixed read-out time window.

Because they remain detectable in the body for decades after the onset of infection, the biomarkers most commonly employed in commercial HIV tests are anti-gp41 antibodies^17,18^. We thus selected the detection of these antibodies as our test bed. To do so we identified from the literature three short gp41 epitopes that range in immunogenicity from high to effectively nonexistent (Table 1).

**Table 1:** The recognition elements employed.

Epitope	Sequence (reported epitopes in bold)	Immunogenicity	Ref.
4B3	LWG**CSGKLVC**TT	High	^18^
2F5	ELL**ELDKWA**SLWNC	Low	^19^
4E10	**NWFDIT**NWLWYIKKKK	Near zero	^20^

**Fig. 3:**
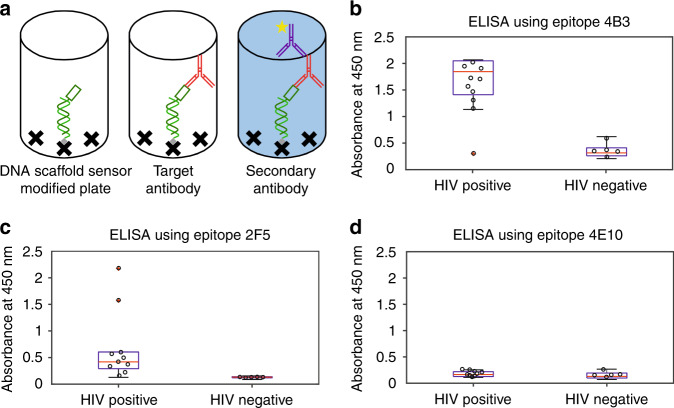
We validated the immunogenicity of three gp41 epitopes using authentic patient samples using a novel ELISA approach that closely mimics the architecture of E-DNA sensors. **a** The ELISA employs a biotin-modified DNA anchor strand hybridized with the complementary PNA-epitope chimera and attached to a streptavidin-coated 96-well microplate. The binding of target antibody is then quantified using a horseradish peroxidase (HRP)-modified secondary antibody that generates a colorimetric signal. The resulting ELISA measurements confirm prior literature reports that: **b** epitope 4B3 is highly immunogenic (nine of ten putatively positive samples reacted, as was also true for the commercial HIV test), **c** epitope 2F5 is weakly immunogenic (only two strongly reacting samples (red)), and **d** epitope 4E10 is effectively nonimmunogenic.

To ensure that conjugation of the epitope to the PNA does not affect immunogenicity, we characterized each epitope using a novel ELISA format that mimics the architecture of the scaffold sensor by replacing the thiolated strand used to anchor the scaffold to the electrode with a biotinylated strand that can be attached to a streptavidin-coated ELISA plate (Fig. 3). We hybridized this to the same PNA-epitope chimera employed in our E-DNA sensors (see Fig. 3a) and used this to perform a standard ELISA. Testing our three epitopes against the same commercially sourced HIV-positive and HIV-negative patient samples described above, we confirmed the prior literature claims. Specifically, nine out of ten HIV-positive samples (the outlier being the sample flagged as negative by the commercial ELISA and lateral flow) proved positive against the 4B3 epitope and all five of the five HIV-negative samples were negative (Fig. 3b), confirming this epitope’s high immunogenicity^19^. In contrast, only two out of ten HIV-positive were positive against epitope 2F5 (including, as expected, all of the known HIV-negative samples), illustrating the lower immunogenicity of the epitope (Fig. 3c)^20^. Finally, the 4E10 epitope, which is effectively nonimmunogenic, did not produce a statistically significant signal change for any of our samples (Fig. 3d) ^21^.

**Fig. 4:**
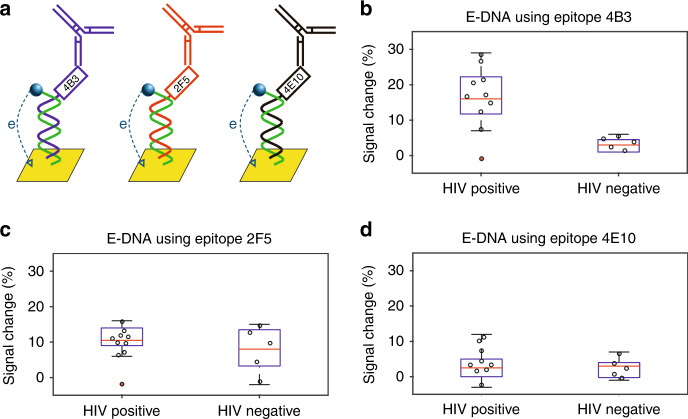
When challenged against our test-bed sample set E-AB sensors achieve the same clinical sensitivity as the commercial tests. To test the clinical performance of E-DNA scaffold sensors we challenged sensors presenting **a** high, low, and no immunogenicity gp41 epitopes against patient samples. Ten of these commercially sourced samples were putatively HIV-positive and five negative. **b** Consistent with the high immunogenicity of the 4B3 epitope, sensors presenting it correctly detected nine out of ten putatively HIV-positive samples (again the missing patient is the same flagged negative by commercial ELISA) and five out of five HIV-negative ones. As was true for the equivalent ELISA and commercial tests, one putatively positive sample did not cause any significant signal change. **c** Sensors presenting the weakly immunogenic 2F5 epitope, in contrast, failed to flag any of the ten putatively HIV-positive samples as positive (at this serum dilution; see below), while flagging as negative all five of the HIV-negative samples. **d** Finally, a sensor presenting the effectively nonimmunogenic 4E10 epitope did not respond to any of the 15 samples.

To validate the clinical performance of E-DNA scaffold sensors (Fig. 4a), we challenged them with the patient samples validated above. The results obtained using sensors employing the 4B3 epitope paralleled exactly those obtained using both commercial tests and the novel ELISA we developed. Specifically, it detected anti-4B3 antibodies in nine of our ten HIV-positive samples (with the outlier being the same sample flagged negative by ELISA and lateral flow) and in none of the five known HIV-negative samples (Fig. 4b). And as was true of the novel ELISA results, we did not detect anti-4E10 antibodies in any of the 15 negative or positive samples (Fig. 4d). However, while the results obtained using sensors employing the 4B3 and 4E10 epitopes paralleled those of the equivalent ELISAs, at this serum dilution (1:80 in PBS) the E-DNA sensors employing the 2F5 epitope failed to produce a positive response against either of the two samples positive for such antibodies in the equivalent ELISA (Fig. 4c).

The signal change obtained with scaffold sensors is small, but it is easily comparable with those of other, established techniques and is more than sufficient to achieve diagnostic relevance. For example, when challenged with HIV-positive samples, the average signal change seen for the 4B3 epitope is approximately 15%, which compares favorably the typical relative signal change seen in the widely clinically employed fluorescence polarization assay^22^. To further demonstrate the effectiveness of E-DNA scaffold sensor as serological test, we performed a receiving operator characteristic curve (shown in Fig. 4S), which place it in between ELISA and lateral flow immunoassays (ROC area: ELISA 0.96, E-DNA scaffold sensor 0.92, lateral flow immunoassay 0.90). Given this, the clinical sensitivity of the E-DNA scaffold sensor is better than that of lateral flow immunoassays, which, as the dominant point-of-care serological test on the market today, represents the direct competitor for E-DNA sensors.

**Fig. 5:**
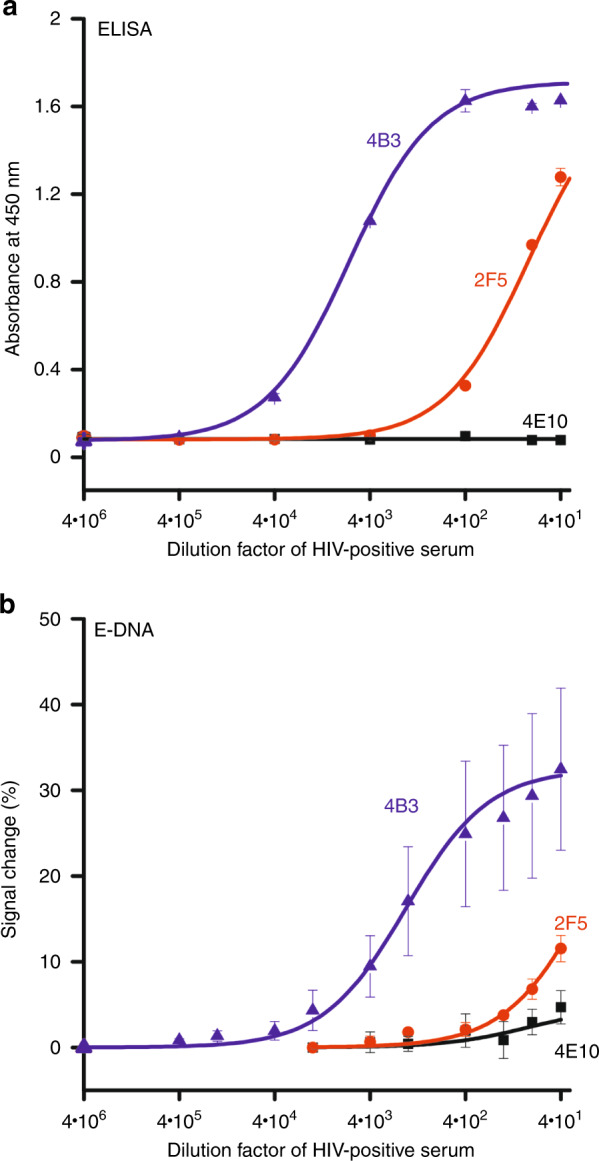
A comparison of the analytical performance of ELISAs and the equivalent E-DNA sensors indicates that, due to the amplification provided by enzymatic signal generation, the detection limits of the latter are an order of magnitude lower than those of the former. The data shown are averaged results obtained using either three wells (for ELISA) or three independently fabricated electrodes (for E-DNA sensors) for each sample dilution, with the error bars representing standard deviations of these measurements. **a** ELISA results: epitope 4B3 produced a useful dynamic range (defined here as the transition from 10 to 90% of the saturated signal) starting at a titer of 1:72,000 and epitope 2F5 a useful dynamic range starting at a titer of 1:960. A sensor displaying epitope 4E10, in contrast, did not respond at any dilution. **b** E-DNA results: epitope 4B3 produced a useful dynamic range starting at titers of 1:14,400 and epitope 2F5 at a titer of 1:40, indicating the ability of the E-DNA scaffold sensors to detect also anti-2F5 antibodies albeit only at higher serum concentrations than those employed above (Fig. 3). A sensor employing 4E10 did not respond at any dilution. The variability in the E-DNA sensor response at low dilution is due to sensor variation seen at high concentrations of serum proteins. Due to the five washing steps (using PBS + 0.05% tween20) they employ, ELISAs are more resistant to such effects.

To determine whether poor analytical sensitivity (i.e., a poor “molar” detection limit) is the reason the scaffold sensor falsely assigned the two samples positive for anti-2F5 antibodies by ELISA as negative, we compared the analytical performance of all three sensors against the equivalent ELISAs. That is, we titrated the three E-DNA sensors with increasing concentrations of the most reactive HIV-positive sample for epitope 2F5 (UID: 403384) while keeping the overall serum concentration fixed at 1:40 via dilution with the appropriate amounts of HIV-negative serum and PBS. For the ELISA the high immunogenicity of epitope 4B3 leads to useful dynamic range (defined as the transition from 10 to 90% of the saturating signal) starting at a titer (dilution) of 1:72,000. The low immunogenicity of epitope 2F5, in contrast, leads to a useful dynamic range beginning at a titer of 1:960 (assuming this epitope saturates at the 1.63 absorbance corresponding to saturation with epitope 4B3) and no significant signal is seen for the nonimmunogenic 4E10 epitope at any dilution (Fig. 5a). The E-DNA scaffold determinations paralleled those seen by ELISA, but with the response curves shifted to higher concentrations. Specifically, the sensor displaying epitope 4B3 exhibited a useful dynamic range starting at a titer of 1:14,400, the sensor displaying epitope 2F5 exhibits a useful dynamic range (assuming it saturates at a signal change of 32% as for epitope 4B3) starting at a titer of 1:40, and the sensor displaying epitope 4E10 did not produce a significant signal change at any dilution (Fig. 5b). And while these results confirm that, due to their enzymatic amplification and numerous wash steps, ELISAs provide better limits of detection, they also demonstrate the ease with which E-DNA sensors can produce quantitative, clinically relevant results that parallel those of ELISAs. And that they can do so in just minutes and using only a single operator step.

## Discussion

Here we demonstrate that E-DNA scaffold sensors can compete with commercial ELISAs and lateral flow assays in terms of clinical sensitivity and specificity. Specifically, our anti-HIV-antibody-detecting E-DNA sensors achieved the required 90% sensitivity and 100% specificity (using this set of samples), in quantitative detail, in two or fewer steps, in less than 12 min, and without requiring a fixed read-out time window (Table 2). And while ELISAs are *analytically* more sensitive (i.e., can detect at lower titers), this does not improve their clinical performance for our test set. Their improved detection limits also come at a significant cost in terms of time, workflow and equipment overhead that renders them ill-suited to application at the point of care. Given the value of reducing the time to diagnosis for sexually transmitted diseases to fit a clinical visit^23^, the E-DNA platform’s improved speed could significantly speed up diagnosis and the treatment initiation, without relying on follow-up visits that often do not occur.

**Table 2:** Comparison of the three technologies.

Parameter	E-DNA	ELISA	LFIA
Number of steps	1–2	~20	1–2
Time to answer	10 min	>150 min	10−12 min
Total cost	N/A^a^	~$488 (+plate reader)	~$26
Cost per sample	N/A^a^	~$5	~$26
Clinical sensitivity^b^	90%	90%	90%
Clinical specificity^b^	100%	100%	100%

^a^It is difficult to evaluate the unit cost for E-DNA scaffold sensors since we currently fabricate them in small batches by hand. Nevertheless, we believe that a E-DNA scaffold sensor for point-of-care use today would cost between $6 and $16 (a screen printed gold electrode costs between $4 and $14, while the reagents would cost approximately $2 per epitope) plus a portable potentiostat, which we showed can cost less than $80

^b^Per the results presented here

As a potential point-of-care technology the E-DNA scaffold platform also compares favorably to lateral flow immunoassays, matching them in terms of ease of use and surpassing them in terms of the clinically relevant information they provide. Specifically, both technologies achieved the same clinical specificity and sensitivity as the commercial ELISA and they have all the requirements for point-of-care deployment being easy to use (both requiring one or two steps) and rapid (10 min). In contrast to lateral flow assays, however, E-DNA sensors are quantitative. Current lateral-flow assays for HIV diagnosis produce only a binary “yes/no” output, which, although useful in assessing the presence or absence of a particular condition (e.g., pregnancy) by a nonspecialized user, can be problematic to use in busy environments since, as noted above, they require the user to read the test at an exact time (too early can produce false negatives; too late false positives). The ability of E-DNA scaffold sensors to quantitatively measure antibody levels not only provides a more precise picture of the patient condition (i.e., clarifying when the patient became infected or, in the case of curable or self-limiting diseases, if it is an active or past infection), but also enhances the clinical specificity defining a cut-off value to ease the diagnosis of uncertain samples, which could generate false results using the subjectivity of lateral flow immunoassays (i.e., different users may interpret the same faint band differently). Unlike lateral flow assays, E-DNA sensors are also easily multiplexed^14^, which is of value because the detection of multiple biomarkers increases clinical sensitivity^24^ and can improve on the identification of proper treatment (e.g., people infected by one sexually transmitted disease have high chances to be co-infected with others)^25^. E-DNA scaffold sensors, which can work in a multiplexed microchip format, thus appear to hold significant advantages over lateral flow immunoassays, which per design and per ease of use (reading a barcode-like test without an automated reader would be challenging even for specialized personnel) cannot integrate more than a few test lines^26^.

## Experimental section

We obtained dual HPLC-purified and lyophilized DNA from Biosearch, Inc. (USA) and epitope-PNA chimeras from PNABIO (USA). The relevant sequences are presented in the Supplementary Information. We purchased gold disk electrodes (2.0 mm diameter), fritted Ag/AgCl electrodes, and platinum wires from CH Instruments, Inc. (TX, USA). We bought streptavidin-coated plates from Thermofisher (15124), while the rest of reagents from Sigma Aldrich including anti-Human IgG (Fc specific) peroxidase antibody (A0170), bovine serum albumin (A3059), and 3,3′,5,5′-Tetramethylbenzidine (T0440). Bioreclamation, Inc. (USA) provided all the patient samples (untreated human serum) used in our experiments. These included authentic patient samples collected from both males and females and across a range of ethnicities (Caucasian, Asian, Hispanic, African-American) and ages (ranging from 18 to 65 years). A complete list can be found in the Supplementary Information.

E-DNA scaffold sensors require linear epitopes to maximize the signal-to-noise ratio. To validate the immunogenicity of potential epitopes, we designed a novel ELISA that uses the epitope-PNA chimeras and a biotinylated DNA anchor that recreates the scaffold employed in the E-DNA sensor on an ELISA plate. To do so, we first washed the streptavidin-coated plate three times using 1× PBS with 2% BSA and 0.05% Tween20 (washing buffer), then we incubated each well with 100 µL of 50 nM of the biotinylated DNA anchor diluted in PBS for 2 h and then washed the plate three times with washing buffer. Following this we incubated each well with 100 µL of 500 nM epitope/PNA chimera solution for 1 h and then washed the plate three times with washing buffer. Next, we incubated each well with 100 µL of serum samples diluted 80 times in the washing buffer for 1 h followed by washing seven times with the washing buffer. We then incubated each well with 100 µL of a solution of peroxidase-modified anti-human antibody (diluted 15,000 times in washing buffer) before washing the plate seven times with washing buffer. Finally, we placed 100 µL of the TMB substrate in each well and incubated for 15 min. The addition of 100 µL of 2 M sulfuric acid stops the reaction and allows the reading using 450 nm absorption wavelength. We performed all incubations with a gentle shaking at room temperature. For washing we used a squeeze bottle.

We prepared E-DNA scaffold sensors following the protocol previously described with minor changes on the PNA incubation buffer and the parameters for the electrochemical detection^14^. Before modifying the electrodes, we prepared them by performing mechanical polishing (using first 1 μm diamond and then 0.05 μm aluminum oxide slurries) and electrochemical cleaning (through successive scans in 0.5 M NaOH, 0.5 M sulfuric acid and 0.1 M sulfuric acid 0.01 M KCl). Meanwhile, we reduced 1 μL of E-DNA anchor (100 μM in ultra-pure water) for 1 h in the dark at room temperature using 5 μL of 10 mM tris(2-carboxyethyl)phosphine hydrochloride (Molecular Probes, Carlsbad, USA). After this reducing step, we diluted the E-DNA anchor solution to 25 nM using PBS. We incubated the clean electrodes in the E-DNA anchor solution for 1 h at room temperature and then in a solution of 3 mM 6-mercapto-1-hexanol overnight at 4 °C. We confirmed the modification of the electrode surface performing square wave voltammetry using 60 Hz frequency, 25 mV amplitude, and a potential window of 0 to −500 mV in PBS. We then incubated the electrode in a solution of 100 nM PNA in PBS with 500 µM EDTA for 2 h (PNA-Bio, the company providing the PNA molecules, recommends the use of EDTA to promote the hybridization between PNA and anchor strand). We confirmed the hybridization repeating the SWV scan. Having the DNA anchor a beacon shape (thus placing the methylene blue in close contact with the electrode surface), the hybridization of the PNA induces the opening of the beacon generating a dramatic change in the electrochemical signal (−75% signal change). For the electrochemical measurement of patient samples, we first run a scan in the negative serum diluted 1:40 in PBS to obtain a baseline, then we added the same volume of 1:40 dilution of the patient sample in PBS. For the sample measurements, we run SWV scans using a frequency of 5 Hz and an amplitude of 50 mV (parameters that we obtained from the optimization described in the supporting information).

A standard method to assess the ability of a diagnostic test to discriminate between positive and negative samples is receiver operating characteristic curve (ROC curves, Fig. 4S). These are plots of sensitivity (the true positive rate) against specificity (the false positive rate) at various threshold values. To calculate the area under our ROC curves for inter-approach comparison, we used the software Origin(Pro), Version Number (e.g. “Version 2019b”) (OriginLab Corporation, Northampton, MA, USA).

## Supplementary information

Supporting Information
