# Supplementary material for: E-DNA scaffold sensors and the reagentless, single-step, measurement of HIV-diagnostic antibodies in human serum
Source: Microsyst Nanoeng. 2020 Mar 23;6:13. doi: 10.1038/s41378-019-0119-5 (PMC8433188; doi:10.1038/s41378-019-0119-5)
Supplement: Supplementary file 1 — Supporting Information [file 41378_2019_119_MOESM1_ESM.docx]

Supporting Information

**E-DNA scaffold sensors and the reagentless, single-step, measurement of HIV-diagnostic antibodies in human serum**

Claudio Parolo^†^, Ava S. Greenwood^†^, Nathan E. Ogden^‡^, Di Kang^†^, Chase Hawes^†^, Gabriel Ortega^†^, Netzahual-cóyotl Arroyo-Currás^¥^, Kevin W. Plaxco^†§^*

†Department of Chemistry and Biochemistry,

‡Department of Material Science

§Interdepartmental Program in Biomolecular Science and Engineering

University of California, Santa Barbara,

Santa Barbara, CA 93106, United States

¥ Department of Pharmacology and Molecular Sciences, Johns Hopkins School of Medicine, Balti-more, MD 21205, United States

N**ucleic acid and epitope sequences**

| **Name** | **Sequence** |
| --- | --- |
| Electrochemical DNA scaffold | 5'-C6-Thiol-G CAG TAA CAA GAA TAA AAC GCC ACT G-MB-3' |
| Biotinylated DNA scaffold | 5'-Biotin-G CAG TAA CAA GAA TAA AAC GCC ACT G-3' |
| PNA epitope 4B3 | LWGCSGKLVCTT–CAG TGG CGT TTT ATT CTT GTT ACT G |
| PNA epitope 2F5 | ELLELDKWASLWNC-CAG TGG CGT TTT ATT CTT GTT ACT G |
| PNA epitope 4E10 | NWFDITNWLWYIKKKK-CAG TGG CGT TTT ATT CTT GTT ACT G |

**Patient list (obtained from Bioreclamation, unless otherwise stated):**

HIV-positive UID: 401352, 200760, 401358, 401366, 277383, 407502, 101818, 403390, 403384, 398785

HIV-negative UID: 479966, 318961, 46403, 455262 + HIV normal serum from Millipore (S1-100ML)

**Electrochemical characterization**


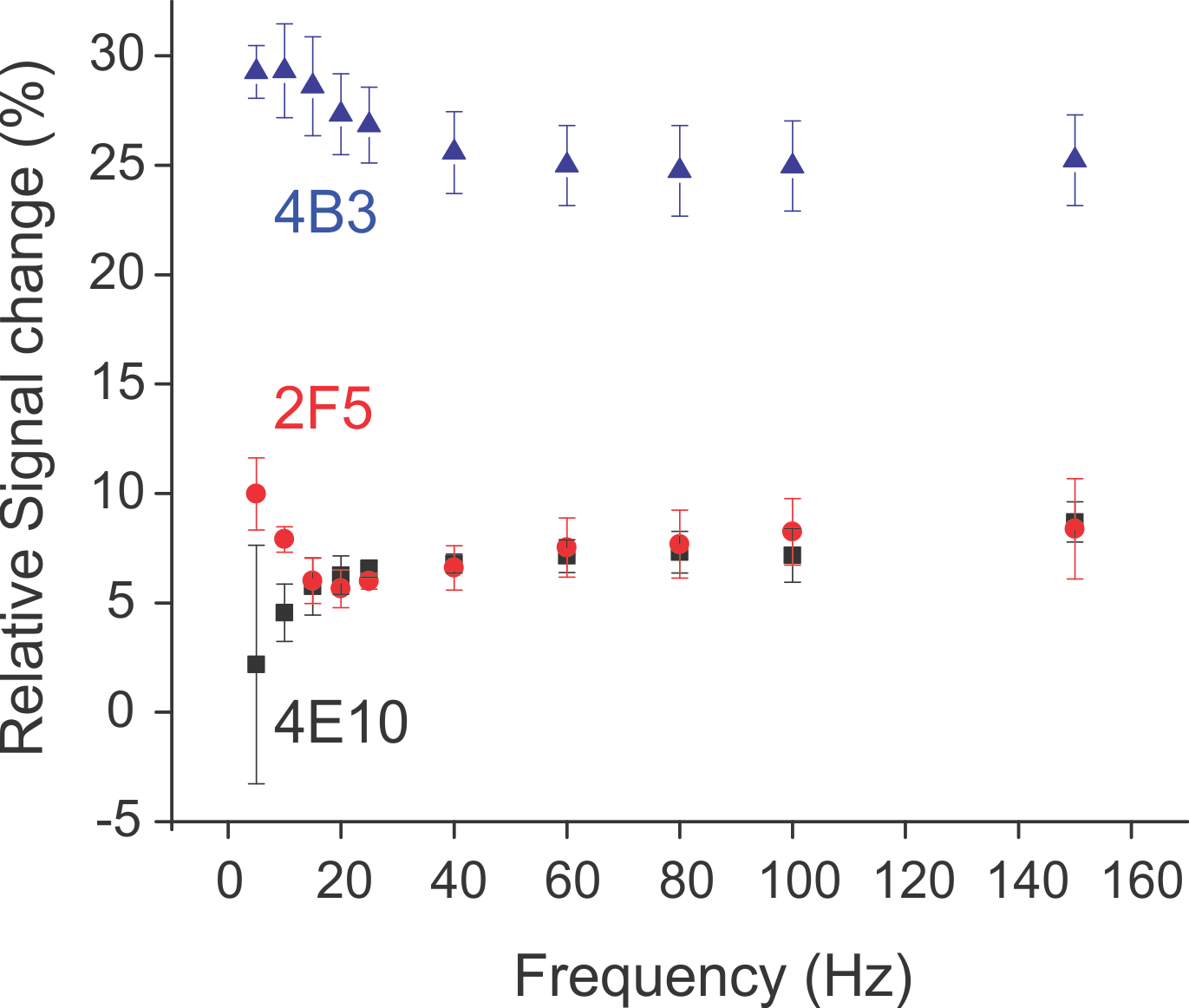


Figure 1S: In order to maximize the performance of E-DNA sensors it is important to evaluate how they respond at different electrochemical parameters.^1^ The graph shows how the relative signal (one less than the ratio of the signal seen in the absence of target derived from measurements of a pool of healthy human serum to the signal seen in the presence of the specific antibody derived from measurements using a pool of HIV-positive serum samples) varies depending on the sampling frequency, using a constant 50 mV amplitude. At low frequencies (5-10 Hz) both immunogenic epitopes 4B3 and 2F5 shows an increment in the signal change, while the non-immunogenic epitope 4E10 does not.


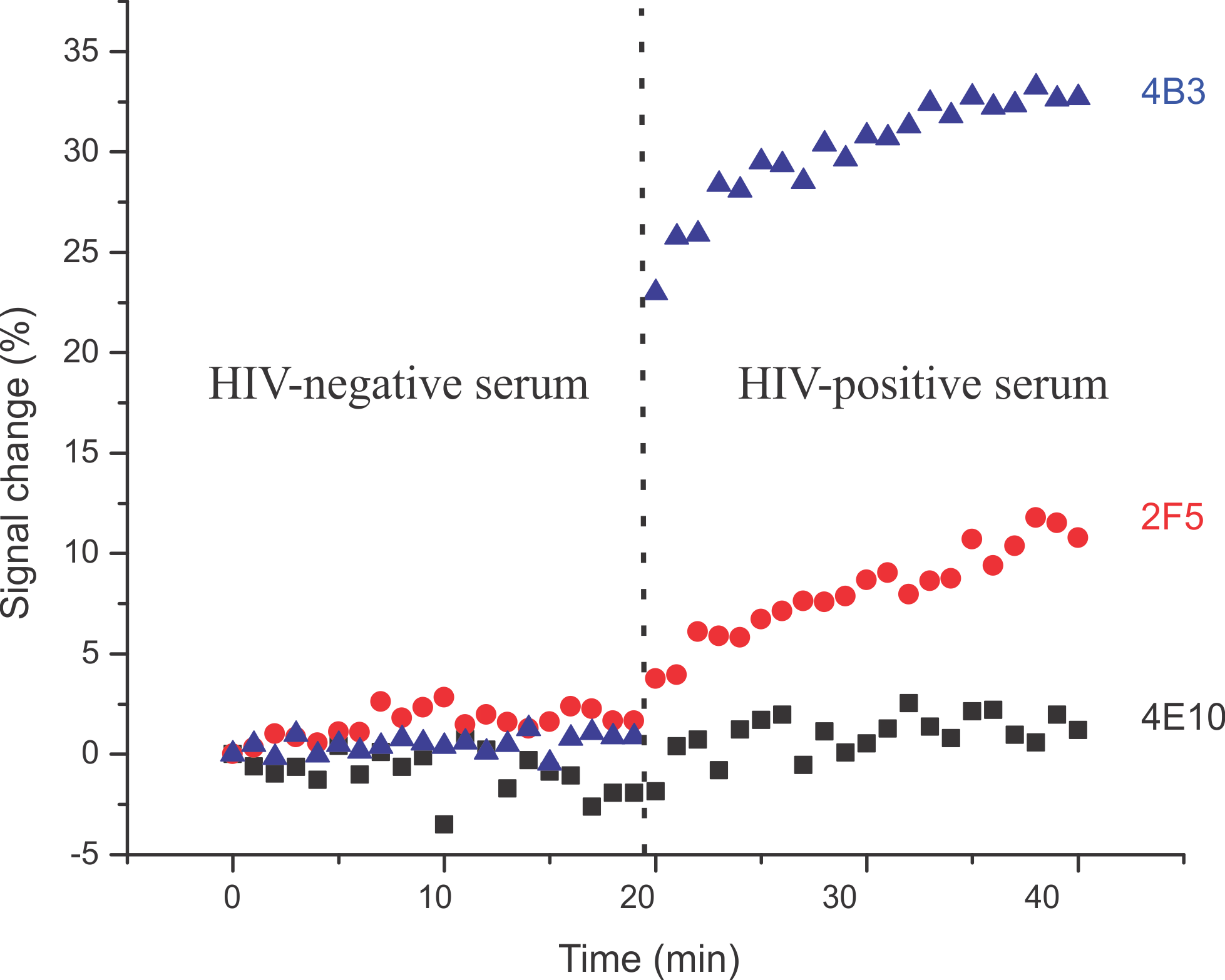


Figure 2S: We also characterized the kinetics of E-DNA scaffold sensors. The graph shows the response of E-DNA scaffold sensors in the presence of both HIV-negative and HIV-positive serums. While for HIV-negative serum the three E-DNA scaffold sensors do not produce any significant signal change over 20 minutes; once we add the HIV-positive serum we observe an almost immediate signal change which plateaus approximately after 10 minutes.

**Lateral Flow immunoassays**


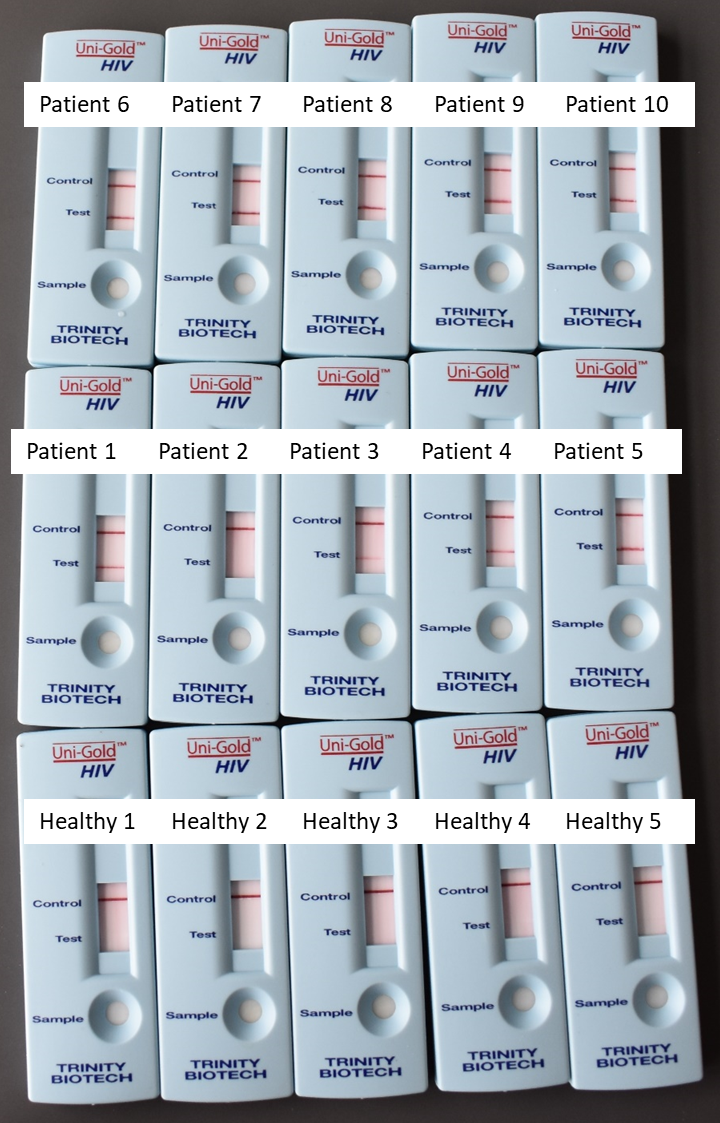


Figure 3S: We tested all samples using commercial lateral flow immunoassays to evaluate how the gold standard for point-of-care serological test performs. The 5 healthy patient samples did not produce any line in the test area of the strip, making for a very good clinical specificity. The 10 HIV-positive patient samples instead produced one allegedly false negative (Patient 2 – the same patient came out as false negative using both ELISA and E-DNA scaffold sensors as well) and one “possibly unclear” test (Patient 3), due to a faint test line.

**Receiving operating characteristics**


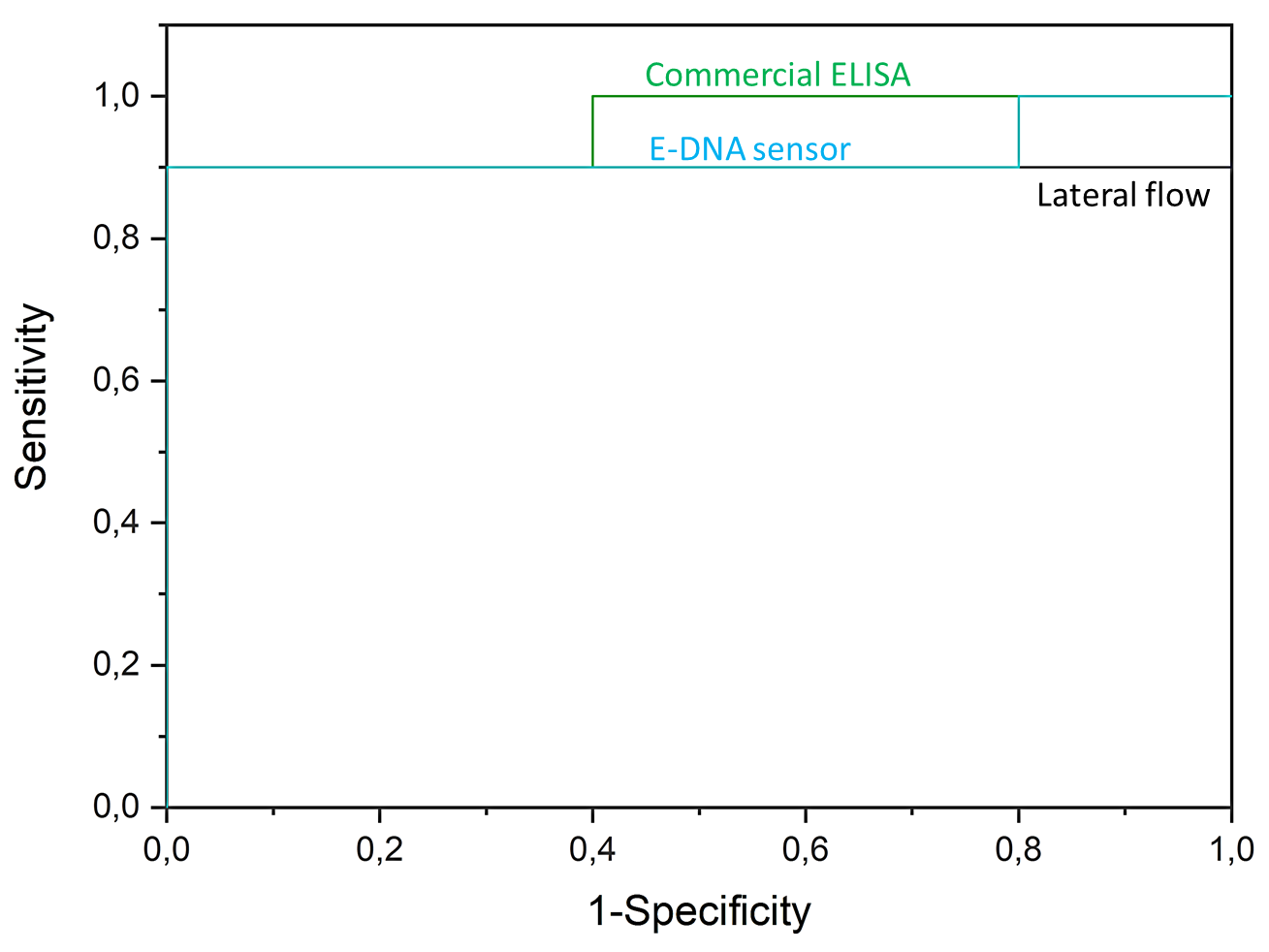


Figure 4S: The graph shows the receiving operating characteristics for commercial ELISA, commercial lateral flow and E-DNA scaffold sensor. As expected commercial ELISA proved to be the most sensitive and specific techniques (area under the curve of 0.96 and asymptotic probability of 0.00485), while E-DNA scaffold sensors did slightly better (area under the curve of 0.92 and asymptotic probability of 0.01011) than lateral flow immunoassays (area under the curve of 0.90 and asymptotic probability of 0.01431). This result shows how E-DNA sensors represent a valid alternative to carry out serological measurement at the point of care.

REFERENCES

(1) Dauphin-Ducharme, P.; Plaxco, K. W. Maximizing the Signal Gain of Electrochemical-DNA Sensors. *Anal. Chem.* **2016**, *88* (23), 11654–11662. https://doi.org/10.1021/acs.analchem.6b03227.
